# Neurological or Psychiatric Disorders After Dengue Fever

**DOI:** 10.1001/jamanetworkopen.2024.10075

**Published:** 2024-05-07

**Authors:** Hong-Ci Lin, Hsueh-Pu Chou, Yung-Chih Chiang, Renin Chang, Yao-Shen Chen, Yu-Chung Juan

**Affiliations:** 1 Department of Medical Education and Research, Kaohsiung Veterans General Hospital, Kaohsiung, Taiwan; 2 Department of Emergency Medicine, Kaohsiung Veterans General Hospital, Kaohsiung, Taiwan; 3 Department of Orthopedics, E-Da Hospital, I-Shou University, Kaohsiung, Taiwan; 4 School of Medicine, College of Medicine, I-Shou University, Kaohsiung, Taiwan; 5 Department of Psychiatry, Kaohsiung Veterans General Hospital, Kaohsiung, Taiwan; 6 Institute of Medicine, Chung Shan Medical University, Taichung, Taiwan; 7 Department of Administration, Kaohsiung Veterans General Hospital, Kaohsiung, Taiwan; 8 Department of Neurosurgery, China Medical University Hospital, Taichung, Taiwan

## Abstract

This cohort study investigates the association between dengue fever and risk of neurological and psychiatric disorders among adults in Taiwan.

## Introduction

Dengue fever, which has an estimated annual infection rate of up to 390 million, mainly affects Central and South America and Southeast Asia.^1,2^ Apart from possible direct invasion of dengue fever virus to the neurological system, the disease has been reported to be associated with neurological complications probably attributable to autoimmune reactions and metabolic alterations.^3^ Several studies^4,5^ have found an association between dengue fever and neurologic and psychiatric disorders, including anxiety and depression symptoms. This retrospective nationwide cohort study attempted to further scrutinize the association between dengue fever and risk of subsequent neurological or psychiatric complications.

## Methods

This cohort study, conducted in 2022, adhered to the STROBE reporting guidelines. Institutional review board approval was secured from the E-Da Hospital of Taiwan, which waived informed consent requisites owing to the anonymization of personal information.

Using data from the National Health Insurance Research Database, which covers 99% of the Taiwanese population, the research enrolled patients diagnosed with dengue fever from 2000 to 2015 who were propensity score matched at a 1:4 ratio with a comparator group without dengue fever based on various demographic and health factors. End points were the appearance of any outcome of interest, end of the study (ie, end of 2017), and death. The primary objective was to investigate whether dengue fever was associated with increased risk of neurological or psychiatric disorders. We excluded patients with preexisting conditions or who were younger than 18 years. The study used *International Classification of Diseases, Ninth Revision *(*ICD-9*) and *International Statistical Classification of Diseases and Related Health Problems, Tenth Revision *(*ICD-10*) codes to identify relevant diagnoses and Cox proportional hazards regression models for analysis.

## Results

A total of 48 884 adults with dengue fever (26 415 male [54.04%]; mean [SD] age, 43.08 [16.54] years) and 195 536 individuals without the diagnosis (105 660 male [54.04%]; mean [SD] age, 43.08 [16.54] years) were identified (Table 1). Adjusted hazard ratios (aHRs) showed an increased risk for neurological and psychiatric disorders in the group with vs without dengue fever, including Guillain-Barré syndrome (1.10; 95% Cl, 1.03-1.18), myoneural junction disease (1.15; 95% CI, 1.08-1.23); Parkinson disease (1.44; 95% CI, 1.12-1.86); dementia (1.23; 95% CI, 1.07-1.41), and psychotic, mood, and anxiety disorders (1.13; 95% CI, 1.08-1.19). No significant difference was observed in the risk of intracranial hemorrhage or ischemic stroke (Table 2). Subgroup analysis based on age reinforced main findings, with aHRs showing an increased risk of composite psychiatric disorders after dengue infection for all age groups (18 to 30 years: 1.14; 95% CI, 1.05-1.24; >30 to 60 years: 1.07; 95% CI, 1.02-1.12; >60 years: 1.22; 95% CI, 1.13-1.31), while a significantly increased risk of neurological disorders was observed in individuals older than 60 years (aHR, 1.17; 95% CI, 1.07-1.28). Sex subgroup analysis revealed a universally higher risk of neurological and psychiatric disorders in the dengue fever group (Table 2).

**Table 1.** Baseline Characteristics of the Study Population

Characteristic	Patients, No. (%) (N = 244 420)	
	No dengue (n = 195 536)	Dengue (n = 48 884)
Age, y		
Mean (SD)	43.08 (16.54)	43.08 (16.54)
18 to 30	53 208 (27.21)	13 302 (27.21)
>30 to 60	105 986 (54.20)	26 497 (54.20)
>60	36 342 (18.59)	9085 (18.58)
Sex		
Female	89 876 (45.96)	22 469 (45.96)
Male	105 660 (54.04)	26 415 (54.04)
Medication		
Aspirin	5756 (2.94)	1601 (3.28)
Colchicine	1842 (0.94)	574 (1.17)
Metformin	7115 (3.64)	2086 (4.27)
Plavix	524 (0.27)	170 (0.35)
Statin	9301 (4.76)	2657 (5.44)
Ticlopidine	184 (0.09)	51 (0.10)
Warfarin	341 (0.17)	91 (0.19)
Follow-ups per year, mean (SD)		
Neurology outpatient department visit	0.49 (3.99)	0.53 (3.98)
Psychiatry outpatient department visit	0.05 (0.99)	0.09 (2.52)
Comorbidity		
Cancer	7513 (3.84)	1652 (3.38)
Chronic kidney disease	2994 (1.53)	786 (1.61)
Coronary artery disease	12 282 (6.28)	2983 (6.10)
COPD	11 484 (5.87)	2764 (5.65)
Diabetes	19 095 (9.77)	4900 (10.02)
Heart failure	3001 (1.53)	643 (1.32)
Hyperlipidemia	30 449 (15.57)	7755 (15.86)
Hypertension	36 279 (18.55)	8926 (18.26)
PAOD	1199 (0.61)	299 (0.61)
Sleep apnea	875 (0.45)	207 (0.42)
Neurological or psychiatric condition	20 447 (10.46)	5218 (10.67)
Neurological condition	7004 (3.58)	1759 (3.60)
Encephalitis	73 (0.04)	22 (0.05)
Guillain-Barré syndrome	20 (0.01)	4 (0.01)
Intracranial hemorrhage	437 (0.22)	116 (0.24)
Ischemic stroke	1878 (0.96)	391 (0.80)
Myoneural junction and muscle disease	205 (0.1)	61 (0.12)
Nerve, nerve root, or plexus disorder	4122 (2.11)	1085 (2.22)
Parkinson disease or parkinsonism	269 (0.14)	80 (0.16)
Psychiatric condition	13 443 (6.87)	3459 (7.08)
Dementia	1032 (0.53)	245 (0.50)
Insomnia	4066 (2.08)	1030 (2.11)
Psychotic, mood, or anxiety disorder	7317 (3.74)	1902 (3.89)
Substance use disorder	1028 (0.53)	282 (0.58)

Abbreviations: COPD, chronic obstructive pulmonary disease; PAOD, peripheral arterial occlusion disease.

**Table 2.** Risk of Disorders Among Patients With Dengue Fever

Disorder	Risk in dengue group, aHR (95% CI)^a^	*P* value
Neurological or psychiatric disorder	1.12 (1.09-1.15)	<.001
Neurological disorder	1.12 (1.06-1.18)	<.001
Encephalitis	1.29 (0.97-1.72)	.08
Guillain-Barré syndrome	1.10 (1.03-1.18)	<.001
Intracranial hemorrhage	1.19 (0.97-1.46)	.10
Ischemic stroke	0.96 (0.86-1.07)	.48
Myoneural junction and muscle disease	1.15 (1.08-1.23)	<.001
Nerve, nerve root, or plexus disorder	0.88 (0.30-2.59)	.82
Parkinson disease and parkinsonism	1.44 (1.12-1.86)	<.001
Psychiatric disorder	1.12 (1.08-1.17)	<.001
Dementia	1.23 (1.07-1.41)	<.001
Psychotic, mood, and anxiety disorders	1.13 (1.08-1.19)	<.001
Insomnia	1.10 (1.03-1.18)	<.001
Substance use disorder	1.14 (1.00-1.30)	.05
**Subgroup analyses**		
Ages 18 to 30 y		
Neurological disorder	1.08 (0.91-1.27)	NA^b^
Psychiatric disorder	1.14 (1.05-1.24)	NA^b^
Ages >30 to 60 y		
Neurological disorder	1.07 (0.99-1.14)	NA^b^
Psychiatric disorder	1.07 (1.02-1.12)	NA^b^
Age >60 y		
Neurological disorder	1.17 (1.07-1.28)	NA^b^
Psychiatric disorder	1.22 (1.13-1.31)	NA^b^
Female		
Neurological disorder	1.10 (1.03-1.19)	NA^b^
Psychiatric disorder	1.12 (1.06-1.18)	NA^b^
Male		
Neurological disorder	1.13 (1.05-1.22)	NA^b^
Psychiatric disorder	1.12 (1.07-1.18)	NA^b^

Abbreviations: aHR, adjusted hazard ratio; NA, not applicable.

^a^ The reference group for all outcomes is patients without dengue fever. Adjusted for age, sex, cancer, chronic kidney disease, coronary artery disease, chronic obstructive pulmonary disease, diabetes, heart failure, hyperlipidemia, hypertension, peripheral arterial occlusion disease, and sleep apnea.

^b^ According to the guidelines of Taiwan's National Health Insurance and Research Database, information will be deleted if it has not been assessed within a 2-year time frame, so these data were inaccessible.

## Discussion

This cohort study’s findings support those of previous research suggesting a potential association between dengue fever and neurological and psychiatric complications, especially in individuals older than 60 years. Although prior case reports and smaller studies^4,5^ noted the neurotropic nature of the dengue fever virus, this study stands out due to its extensive scale and use of long-term, comprehensive national health data, which may avoid selection bias inherent in smaller datasets.^6^ The study has several limitations. Diagnoses were based on *ICD* codes, which may be prone to misclassification. The lack of detailed patient information (eg, lifestyle factors and severity of dengue fever) may have influenced results. Furthermore, our findings may not be generalized beyond the Taiwanese population.

This population-based retrospective study provides updated epidemiological evidence for the association of dengue fever with increased risk of neurological and psychiatric disorders. Findings may highlight the need for awareness and monitoring of neuropsychiatric complications in this patient population. However, further research is necessary to understand mechanisms behind these associations.

## Notes

Supplement.
Data Sharing Statement
