# Supplementary material for: Neurological or Psychiatric Disorders After Dengue Fever
Source: JAMA Netw Open. 2024 May 7;7(5):e2410075. doi: 10.1001/jamanetworkopen.2024.10075 (PMC11077384; doi:10.1001/jamanetworkopen.2024.10075)
Supplement: Supplement. — Data Sharing Statement [file jamanetwopen-e2410075-s001.pdf]

Data Sharing Statement

Lin. Neurological or Psychiatric Disorders After Dengue Fever. *JAMA Netw Open*. Published online May 7, 2024. doi:10.1001/jamanetworkopen.2024.10075

Data
Data available: No

Additional Information
Explanation for why data not available: Summarized individual data are available on request to the corresponding author. The data set used in this study is managed by the Taiwan Ministry of Health and Welfare and, thus, cannot be made available publicly. Researchers interested in accessing this data set can submit a formal application to the Ministry of Health and Welfare to request access (the postal address No. 488, Section 6, Zhongxiao E Rd, Nangang District, Taipei City 115, Taiwan; website: https://dep.mohw.gov.tw/ DOS/cp-2516-3591-113.html).
